# “I Feel Locked Out the Community”: The Experience of Community for Rural-Living Fisters

**DOI:** 10.1007/s10508-025-03293-1

**Published:** 2026-02-05

**Authors:** Jarred H. Martin

**Affiliations:** Department of Psychology, Faculty of Humanities, University of Pretoria, Lynnwood Road, Hatfield, Pretoria, 0002 South Africa

**Keywords:** Fisting, Gay, Rural, Sexual orientation, Kink, Interpretive Phenomenological Analysis

## Abstract

Research has highlighted the important role that communities of kink play in the social and sexual lives of kinksters. Existing literature has not examined the experience of community for rural and remote-living kink-identified individuals, where access to communities, networks of playmates, and spaces for kink may be limited by virtue of social and geographic isolation. The aim of this study was to explore the lived experience of community among self-identified cisgender gay men who engage in fist-fucking and live in rural areas. A qualitative study was conducted with a multi-national sample of 40 fist-fuckers, each of whom participated in an online semi-structured individual interview. Guided by a interpretative phenomenological analysis of the data, six themes were developed: (1) the experience of isolation in the formative journey of self-discovery; (2) geographic, logistical, and financial burdens in accessing community; (3) feelings of (dis)connection from the community and the struggle for kink identity; (4) the absence of community networks and resources in supporting healthcare needs; (5) the opportunities and challenges of online communities and connections; and (6) the lack of communal spaces and local networks of partners in fostering sexual satisfaction. The findings revealed that participants shared lived experiences of personal isolation, sexual frustration, and social exclusion from urban-based fisting communities. For some fist-fuckers, their rural-living circumstances produced not only social and sexual dislocations from their communities but, also, a disconnectedness from their kink identity. For others, the remoteness of their living contexts forged new modes of online community building, strategies for sexual and erotic resilience, and experiences of community.

## Introduction

Communities formed by kink-identified individuals are considered to provide them social as well as sexual networks and spaces within which they can explore and express their alternative sexualities, identities, and practices (Newmahr, 2011; Ortmann & Sprott, 2012). Studies across different kink communities have shown that these affiliative networks play a series of significant roles in the lives of kinksters, particularly those who desire and value community connection. They enable kinksters to establish affirmative meanings around their desires and practices; access resources, knowledge, and safe play spaces; and form connections that foster belonging, facilitate skill-sharing, and offer emotional, psychological, and practical support (Boyd-Rogers & Maddox, 2022; Graham et al., 2015; Pohtinen, 2019; Sprott et al., 2019). However, existing research has largely focused on the urban settings in which these communities have been traditionally localized and organized. A consequence of this has not only been the relative neglect of the identities, practices, and relationships of kinksters living outside of urban centers and, more specifically, those living in rural and remote areas, but also the persistence of a metronormative account of community in the lives of kinksters.

This study focuses on the kinked community of gay men who fist-fuck and specifically provides a qualitative interpretative phenomenological analysis (IPA) of the experience of community for fist-fuckers who live in rural and remote areas. Fist-fuckers are particularly valuable for exploring experiential questions of community and rurality because so much of their modern subcultural history and lore is rooted in the urban sexual geographies of the 1970s, particularly in the bars, backrooms, and bathhouses frequented by gay leathermen and fetishists in cities like San Francisco, Los Angeles, and New York (Herrman, 1991; Norton, 2006; Rubin, 1997). Furthermore, contemporary research has highlighted the significance of community membership and interaction for fist-fuckers. Martin (2025a) has found that communal ties and engagement among fisters plays a formative role in their kink identity, while Martin (2025b) has showed that accessible in-person communities of playmates also transmit practical skills, knowledge, and norms for safe and satisfying fist-play.

### Kink and Community

The term kink refers to a wide range of erotic interests, identities, practices, and relationships that fall outside mainstream sexual norms (Vivid et al., 2020; Williams & Sprott, 2022). Although some studies refer to “*the* kink community” (Nevard, 2019 p. 617; Rehor, 2015, p. 826, emphasis added) to distinguish between those who engage in kink privately and those who participate in social networks, events, and spaces that center around kink (Newmahr, 2010), in practice, no singular, homogeneous kink community exists (Rehor & Schiffman, 2021). Rather, kink comprises “a diverse and evolving field of different (and sometimes overlapping) erotic niches that are often embedded in more and less established subcultural communities, each with its own histories, norms, and values that regulate membership, identification, and practices” (Martin, 2025a, pp. 398–399). Among kink-identified individuals, the idea of community can itself be applied flexibly and unevenly, sometimes referring to “the scene” as a whole and, at other times, to specific networks of kinksters, organizations, meeting spaces, websites, and events (Weiss, 2011). To this end, communities of kink-identified individuals may vary not only in the kink around which they are oriented but, moreover, in their degree of formal organisation and affiliation, the extent of their reach and association (local, regional, and international), the preferred means and frequency of in-person or online interaction, the level of public visibility and political advocacy, as well as member and outsider accessibility.

Early scholarship on kink, albeit with some exceptions (see Weinberg et al., 1984; Weinberg, 1987), did not always center the communal dimensions of kink. According to Weinberg et al. (1984), this may have been an effect of a scientific tradition influenced by atomistic paradigms of individual morality and psychology that dislocated kinksters from their subcultural contexts and, ultimately, theorised their alternative sexualities “as either an abstract problematic or an individualised orientation” (Weiss, 2011, p. 12). More recent research has foreground kink as a social and communal phenomenon, embedded in spaces and networks which shape how it is practiced, (re)interpreted, and sustained over time (Stein, 2021). This work has also highlighted the internal complexities and contradictions of kink communities, including the presence of identity hierarchies and social cleavages that reproduce patterns of kinknormativity and perpetuate exclusions along lines of race and class (Bennett, 2025; Beveney, 2023; Erickson et al., 2022).

The advent of the internet has further transformed modern kink communities by extending their reach beyond geographically bound groups to encompass transnational, digitally connected networks of kinksters (Křivánková, 2024; Simula, 2019; Wignall, 2022, 2023). Nonetheless, as Weinberg (2023) has noted, there remains a significant gap in our understanding of kink communities in the Global South, as most of the existing research has been conducted with samples of kinksters based in Western Europe and North America.

### Fisting and Community

Anal fisting is the solo or (multi-)partnered practice involving the insertion of one or both hands, typically beyond the wrist but no further than the upper arm, through the anus and into the rectum and lower colon. Fist-fuckers describe the experience of fist-play as a fusion of intense sexual and erotic sensation that also produces profound feelings of intimate partner connection and, for some, altered states of consciousness (Brough, 2005; Niederwieser, 2013; Shockey, 2009). In the context of kink, fisting is usually regarded as form of ‘edge play’ because of its physical and psychological intensity, potential for harm, and the high degree of skill, trust, and consensual negotiation required for participation. It is, however, not limited to communities of self-identified fist-fuckers, but has been documented in the play practices of other sexual subcultures, including leatherfolk (Mains, 1991), bears (Moskowitz et al., 2013), BDSM practitioners (Bondage and Discipline, Dominance and Submission, and Sadism and Masochism; Holmes et al., 2018), pigs (Florêncio, 2020), and pups (Wignall et al., 2022). Although the exact number of individuals who identify as fisters is not known, research suggests that fisting remains relatively uncommon among the general population, while some evidence and anecdotal accounts indicate its growing popularity among certain groups of gay men (Prestage, 2002; Rehberg, 2023; Richters et al., 2014).

Some of the earliest research on anal fisting amongst gay men emerged from medical and forensic case studies that identified it as a “sexual activity confined primarily to the homosexual community” (Reay & Eisele, 1983, p. 347; see also Crass et al., 1981; Elam & Ray, 1986; Lowry & Williams, 1983; Reiner, 1984; Shook et al., 1985; Sohn et al., 1977; Spears et al., 1995; Weinstein et al., 1981), with little to no reference to a specific community of fisters. In the few instances where a communal context was acknowledged, fisting was often subsumed within what Katner & Pankey (1987, p. 1071) described as “a number of traumatic practices” that were attributed to the “the organisation of sadomasochistic societies” in the 1970s.

One of the first studies to situate fisting within an organized subculture of fisters (then also known as handballers) was Navin’s (1981) interview-based research with fist-fuckers living in the United States. This work is significant because it identified handballers as a distinct “group … [with] their own special activities” (Navin, 1981, p. 69), that could be distinguished from the broader leather and BDSM community of the time. Moreover, Navin identified the formation of organized associations for fist-fuckers, including the Fist Fuckers of America (FFA) and Mid America Fists in Action (MAFIA) in the US, as well as the Total Ass Involvement League (TAIL), which purportedly had members across the US, United Kingdom, and Australia. In the same year, Suppe’s (1981) reference to the colour-specific “hanky code” employed by fist-fuckers to signal their sexual interests also points to the emergence of shared aesthetic codes used to constitute a recognisable subcultural identity within urban communities of gay men at that time.

Perhaps the most significant contribution to frame fisters as a subcultural community is Rubin’s (1991) ethnographic account of The Catacombs, an underground private communal space that operated in San Francisco between 1975 and 1981. Rubin (1991, p. 226) described The Catacombs as the “Mecca of handballing,” a place where “fisters from all over the Western world made the pilgrimage.” These early fisting spaces brought fist-fuckers together through “a common set of facilities, rules, symbols and emotions,” as Brodsky (1993, p. 246) similarly observed in his description of Mineshaft, a fist-friendly BDSM and fetish club that operated in New York between 1976 and 1985. These accounts underscore the formative role that physical spaces and events for fisters played in the subcultural placemaking of the first fisting communities and in establishing the norms which scaffolded their corpo-erotic codes of desire, practice, and care (Califia, 1994; Fritscher, 1978).

Once limited to niche urban spaces and events like the annual Folsom Street Fair, fisters now congregate through a host of organized fisting communities located in some of the major urban centers in North America, Europe, Australia, and Japan, amongst others. These communities are supported by dedicated organisations, clubs, festivals, and educational workshops tailored for fisters. Online kink-oriented social networking sites such as FetLife also play an important role by hosting fisting-specific groups and discussion forums, alongside more informal digital micro-communities such as “Fisting Twitter”–a loosely connected network of fist-positive X users who share images, tips, memes, and cultural commentary related to fisting. Additionally, mobile applications like Scruff, Recon, and AssPig cater to individuals interested in fisting within gay kink communities, combining dating app functionality with event listings and other fisting-related media.

A recent set of studies with fisters in South Africa have shown that community functions as a vital repository of subcultural norms and knowledge that shapes their skill and identity formation (Martin, 2020, 2022, 2023, 2024, 2025a, 2025b). However, this work has focused exclusively on fisters living and playing in metropolitan areas characterised by networks of closely connected playmates and scenes of in-person play. While these studies are valuable in providing a Southern counterpoint to the dominant body of Western kink research, the reliance on urban-based fisters risks “scaling up urban queer experiences as nationally representative, homogenising non-Western queer sexualities and eschewing […] non-Western queer lives outside of major urban centers” (Cummings, 2024, p. 1). This inadvertently reinscribes urban areas as central and rural areas as peripheral, and repeats long-standing ideas that are believed to distinguish urban and rural life from one another, such as, presence/absence, modern/backward, complex/simple, visible/invisible, connected/isolated, and liveable/unliveable. These distinctions have also shaped how queer lives and kinked sexualities are imagined and represented (Stone, 2018).

### Rurality, Sexuality, and Kink

Since the 1990s, the urban bias in the study of lesbian, gay, bisexual, intersex, transgender, and queer (LGBTIQ +) lives has been increasingly challenged by scholarship that contests the assumption that queer sex, identity, kinship, and community are inherently tied to urban spaces and thus only visible and viable within metropolitan centers (Bell & Valentine, 1995; Binnie & Valentine, 1999; Forstie, 2022; Gray, 2009; Weston, 1995). Halberstam (2005) extended this critique by arguing that studies of queer coming out and life have often been bound to metronormative metanarratives that locate value with/in urbanity, while neglecting and homogenising the suburbs, smaller cities, towns and rural areas where queer lives also unfold. Building on this, Thomsen (2021) has argued for the need to disrupt dominant paradigms of visibility by showing how rural queer life is frequently rendered either non-existent or in need of rescue through urbanisation. Thomsen’s (2021) idea of “visibility interrupted” challenges the assumption that queer identities can only thrive and flourish within urban spaces, and instead points to the ways in which forms of queerness are lived, expressed, and made visible beyond the physical boundaries and the cultural, aesthetic, and commercial forces of the city.

In line with this, studies of rural-living LGBTIQ + individuals have documented how they construct alternative forms of belonging, navigate geographic and social isolation, and develop strategies for identity expression, safety, and social connection outside urban queer enclaves (Gray et al., 2016). However, in the study of alternative sexualities, the intersection of rurality and kink has largely gone unexamined. In Hughes and Hammack’s (2019) study of kink identity narratives among an international sample of 265 people recruited from FetLife, there is only a brief mention of the geographic and political isolation experienced by one rural participant. Similarly, in a study focused on the mental health of kink-oriented individuals (n = 52) living in rural and remote Tasmania, Reynish et al. (2023) also highlighted accounts of isolation. However, this research also found that 75% of participants reported that involvement in a kink community positively impacted their mental health. Despite these insights, significant gaps remain in understanding how rural kinksters seek out and access community, how they navigate their (dis)connectedness from broader kink networks, and how they experience intimacy, care, and support in their everyday lives, especially in contexts where kink is heavily stigmatised (even among LGBTIQ + people) and where kinkphobic violence may be a lived reality.

Examining these questions is essential for developing an account of kink intimacy, desire, and sociality that acknowledges how the spatial and cultural dimensions of rurality inform understandings of community among rural-living kinksters. It also moves towards a more capacious account of community which traces alternative narratives of community for kinksters whose rural lives have, for the most part, been rendered invisible in popular discourse and scholarly research. Specifically, little is known about how rural-living fist-fuckers experience community, forge connections, or navigate the social and material constraints of their environments. This study seeks to address that gap by asking: What is the experience of community for fisters who live in rural and remote areas?

## Method

An exploratory and cross-sectional qualitative research design oriented by an IPA guided the collection and analysis of data for the present study. Phenomenology represents a broad school of thought that, although comprised of diverse philosophical assumptions that underpin different methods of research, principally centers the study of individuals’ immediate lived experience (Moustakas, 1994). It is an approach predicted on Husserl’s (1931) shift away from Cartesian positivist philosophies which scaffolded and reinforced a series of epistemological and ontological binaries underpinning the scientific project including mind/body, subject/object, and self/world. Husserl theorized that we do not live and interpret our bodies, selves, and worlds through a detached deduction localized in one or the other pole of these binaries, but do so within and through our intentional and relational lifeworld of experience. It is the study of this lifeworld that occupies the focus of Husserlian phenomenological study (Vagle, 2018).

### Participants

Recruitment was conducted in three waves using purposive and snowball sampling strategies, led by the researcher between December 2024 and March 2025. Guided by the inclusion criteria for the study, the first phase employed a purposive sampling method to establish an initial pool of eligible participants and referral contacts. The researcher reached out via email and WhatsApp, drawing on prior research with fisters (South Africa) and personal connections formed through events and workshops hosted by community clubs and organisations. This phase resulted in the recruitment of 11 participants who met the study’s inclusion criteria, along with 12 additional contacts who, while not participating themselves, provided referrals to others.

The subsequent two waves relied on successive snowball sampling to expand the recruitment network. Snowball sampling, also known as network or reputational sampling, is particularly effective for reaching hard-to-access populations, such as kinksters in rural and remote areas, by using early contacts and participants as “gatekeepers” who facilitate connections with others who meet the study criteria (Liamputtong, 2007). To facilitate the snowball sampling process, initial contacts were provided with the eligibility criteria for the study and asked to identify individuals within their socio-sexual networks who may be eligible to participate. Rather than directly sharing the contact information of potential referrals with the researcher, the referring participants were instructed to first approach these individuals, inform them about the study, and, if the individuals expressed interest in being contacted, then provide their contact information to the researcher. In this way, thee researcher attempted to ensure that the final enrollment of referred individuals into the study would only be known by the researcher and that referred individuals could consent to being contacted. Through participant referrals, the researcher was introduced to 49 additional individuals, of whom 13 were eligible and agreed to take part in wave two. These participants, in turn, referred the researcher to 35 additional individuals, resulting in the recruitment of 16 more participants in wave three. While Dworkin’s (2024) recommendations guided the aim for a recruitment threshold of at least 25–30 interviews to enhance the prospect of qualitative saturation and maximize the conceptual development of the thematic categories, recruitment continued beyond this threshold until meaning saturation was reached (Hennink et al., 2017). Following Hennink et al.’s guidance, recruitment ceased at 40 participants when no new themes or experiences of community emerged in the data analysis.

Participants were eligible for inclusion in the final sample based on the following criteria: they had to (1) self-identify as gay men, (2) currently or previously engage in fisting as part of their preferred sexual practices, (3) reside in a rural area–defined as outside major urban centers with limited access to kink communities, playmates, events, or resources, (4) be 18 years or older to consent to participate, (5) be willing and able to participate in an online interview, and (6) be fluent or comfortable communicating in English. Lastly, participants needed to identify as fisters and report a subjective affiliation to the fisting community. Those who experienced their participation in fisting solely as an isolated sexual practice without broader meanings or affiliative ties to the/a fisting community were excluded.

The final sample consisted of 40 cisgender gay men living in rural and remote areas in their country of residence. Table 1 summarises the main characteristics of each participant in the sample. Of the sample, 15 participants lived in the US while the majority (25) resided in a range of countries, including Australia (3), New Zealand (3), Germany (3), Japan (3), Brazil (2), Canada (2), the UK (2), Sweden (2), Denmark (1), Norway (1), France (1), South Africa (1), and Namibia (1). Participants’ ages ranged from 25 to 52 years, with a sample average of approximately 38 years. Half of the sample (20) identified as White/European/Caucasian (W/E/C) and the other half (20) identified as a Person of Colour including 5 who described themselves as Black/African/African Diaspora (B/A/AD), 4 Latino/Hispanic (L/H), 3 East Asian (EA, e.g. Chinese, Japanese, Korean), 2 Middle Eastern/North African (ME/NA), 2 Mixed/Multiracial (M/M), 2 Indigenous (In, e.g. First Nations, Native American, Aboriginal, Māori), 1 South Asian (SA, e.g. Indian, Pakistani, Bangladeshi, Sri Lankan), and 1 Southeast Asian (SEA, e.g. Filipino, Thai, Vietnamese, Indonesian, Malaysian).

**Table 1:** Participant characteristics

Participant	Age	Country of residence	Self-identified ethnicity	Years fisting	Preferred position for play	Relationship status	Current frequency of in-person scenes of contact play	Self-described rurality classification	Years in rural residential area
P1*	32	USA	W/E/C	5	Bottom	Single	Monthly	Semi-Rural	8
P2	45	USA	B/A/AD	12	Bottom	Single	Rarely	Rural	5
P3	29	Australia	W/E/C	3	Bottom	Single	Every few months	Rural	1
P4	38	USA	B/A/AD	10	Bottom	Single	Every few months	Rural	2
P5	50	USA	W/E/C	20	Top	Single	Never	Remote Rural	10
P6*	27	Germany	ME/NA	2	Versatile	Single	Weekly	Semi-Rural	3
P7	41	USA	L/H	15	Bottom	Single	Rarely	Rural	4
P8	36	New Zealand	In	7	Top	Casual relationship	Rarely	Rural	6
P9*	34	USA	W/E/C	6	Bottom	Single	Monthly	Remote Rural	12
P10*	48	USA	W/E/C	11	Top	Single	Rarely	Rural	18
P11*	30	Sweden	W/E/C	4	Bottom	Single	Every few months	Rural	5
P12	39	Norway	W/E/C	9	Bottom	Single	Every few months	Rural	9
P13*	52	Denmark	W/E/C	18	Bottom	Single	Rarely	Remote Rural	25
P14*	28	Japan	EA	3	Versatile	Single	Monthly	Rural	25
P15	42	Japan	EA	14	Bottom	Single	Rarely	Rural	10
P16	31	South Africa	W/E/C	5	Bottom	Civil union / partnership (CNM)	Every few months	Remote Rural	2
P17	44	USA	W/E/C	10	Bottom	Single	Rarely	Remote Rural	8
P18*	33	Canada	B/A/AD	4	Versatile	Single	Rarely	Rural	16
P19	37	Australia	SEA	10	Bottom	Single	Every few months	Remote Rural	8
P20*	46	USA	M/M	13	Bottom	Single	Never	Remote Rural	23
P21	35	USA	W/E/C	6	Versatile	Single	Monthly	Rural	3
P22	47	Germany	ME/NA	11	Bottom	Single	Rarely	Rural	8
P23	26	USA	L/H	2	Top	Dating	Every few months	Remote Rural	1
P24*	49	New Zealand	W/E/C	20	Versatile	Married (Open)	Every few months	Semi-Rural	22
P25*	40	USA	W/E/C	12	Bottom	Single	Never	Remote Rural	27
P26	43	USA	B/A/AD	19	Bottom	Single	Monthly	Rural	9
P27*	25	Japan	EA	1	Bottom	Casual relationship	Rarely	Rural	2
P28	51	UK	M/M	21	Bottom	Single	Weekly	Semi-Rural	15
P29*	29	USA	W/E/C	3	Top	Single	Every few months	Rural	12
P30	38	Germany	W/E/C	9	Versatile	Single	Rarely	Rural	4
P31	27	Brazil	L/H	3	Bottom	Dating	Never	Rural	1
P32*	48	Sweden	W/E/C	7	Top	Married (Open)	Never	Remote Rural	19
P33	36	USA	W/E/C	5	Bottom	Single	Every few months	Remote Rural	2
P34	32	Canada	B/A/AD	10	Bottom	Single	Every few months	Rural	9
P35*	31	Australia	W/E/C	2	Bottom	Single	Rarely	Rural	4
P36	50	UK	SA	20	Bottom	Single	Rarely	Rural	14
P37	44	Namibia	W/E/C	15	Bottom	Single	Rarely	Rural	6
P38*	28	France	W/E/C	6	Top	Casual relationship	Every few months	Remote Rural	10
P39	30	Brazil	L/H	5	Versatile	Single	Rarely	Rural	3
P40*	43	New Zealand	In	4	Bottom	Single	Weekly	Semi-Rural	12

^*^Discovered and started fisting while living in a remote-rural, rural, or semi-rural area

Within the sample, levels of experience with fisting ranged from 1 to 21 years, with an average of around 9 years. In terms of preferred play positions, the majority of participants identified as a versatile-bottom or exclusive bottom (i.e., participants who prefer or only take the receptive role during play; 26), followed by 7 who identified as completely versatile (i.e., participants who have a preference to switch or exchange between roles, either within the same play session or in different scenes), and 7 who were versatile-top or an exclusive top (i.e., participants who prefer or only take the insertive role during play). Most participants (33) were single, while the remaining were in casual relationships, dating, in an open marriage, or in civil unions or other partnerships that were consensually non-monogamous.

Participants were asked to self-categorise the rurality of their current living situation according to three descriptive categories developed by the researcher: (1) remote rural: the person feels completely disconnected from kink communities due to extreme geographic distance, no local kink spaces or playmates available, and travel to access kink-related events or partners is rarely feasible; (2) rural: the person experiences difficulty accessing in-person kink communities, some kink-friendly spaces or potential playmates exist but are scarce or require travel, and travelling long distances (typically 1+ hours) is often necessary to participate in kink activities; and (3) semi-rural: has some access to kink communities, either through small local networks or manageable travel to urban areas, barriers still exist (e.g. smaller local scene, discretion concerns), but occasional in-person engagement is possible. Among this sample, 17 participants described themselves as having discovered and started fisting while living in a remote-rural, rural, or semi-rural area, while 23 had begun fisting while living in an urban area or city, before relocating to their current rural-living situation. Using these categories, the majority of the sample described themselves as presently living in a rural area (23), with some living in a remote-rural area (12) and a few in semi-rural settings (5). Among the participants, the current frequency of in-person play varied considerably, with 15 participants indicating they played rarely (i.e., less than once a month), 12 every few months, 5 on a monthly basis, 5 never (due to their geographic isolation), and 3 weekly.

### Procedure

The present study specifically adopted an IPA based on the work of Smith et al. (2009). IPA focuses on the detailed exploration of individuals’ lived experiences, emphasising how they make sense of those experiences within their social and cultural contexts. Thus, with the aim of understanding how rural-living fisters experience community, an IPA design proves usefully attuned to capturing the nuanced, subjective meanings these fisters attach to their everyday sense of social and erotic belonging and connection in contexts where networks of community may be less physically proximate, geographically accessible, or formally structured.

An online individual interview was conducted by the researcher with each participant. Every interview started with general introductions and a reiteration of the aims of the study, the rights of the interviewee, and the ethical obligations of the researchers. The interview then proceeded on to a brief set of socio-demographic questions to build a descriptive profile of each participant and the sample as a whole including their age, country of residence, ethnicity, years involved in fisting, preferred position of play, relationship status, frequency of in-person scenes, the rurality or remoteness of their living situation, and the years spent living in a rural area. Thereafter, the substantive portion of the interview commenced with questions being guided by a semi-structured interview schedule. This portion of the interview consisted of four topics that oriented the line of questioning: (1) the interviewee’s journey into fisting, with a focus on their experiences of entering the kink while living in an urban or rural area; (2) the interviewee’s relationship with and understanding of their kink community; (3) the interviewee’s experience of community while living in a rural and remote area; and (4) a closing question which asked the interviewee to share any further experiences related to the focus of the study that they felt had not been discussed during the interview. On average the interviews lasted 90 min and were audio-recorded for later transcription.

Participants were provided with an information and consent form for the study via email and provided written informed consent to participate. There was no incentive provided for participation and participants were allowed to withdraw themselves at any point of study.

### Analysis

The idiographic orientation of IPA ultimately means that the present study does not endeavour towards making universal or definitive claims about human experience but to give voice to the particularity of lived experience. Nonetheless, Smith et al. (2009, p. 38) assert that “in a good IPA study, it should be possible to parse the account both for shared themes, and for the distinctive voices and variations on those themes.” To this effect, the analysis was conducted with aim of first identifying themes of the experience of community that may be unique to each participant and, thereafter, comparing and contrasting these individual accounts with the aim of developing a thematic picture of shared experiences of community across participants.

Conducted by the researcher and following an IPA (Smith & Osborn, 2003; Smith et al., 1999, 2009), each transcript was loaded into NVivo, read and reread for familiarity, with initial notes made during the rereading. These notes included descriptive, linguistic, and conceptual comments that drew the attention of the researcher to patterns in participants’ language use, points of emphasis, contradictions, and possible underlying meanings embedded in their accounts of community. Emergent themes within a transcript were first identified with codes and then consolidated into interview-specific themes. This process was repeated for each transcript and, once all transcripts had been analysed, superordinate themes across the transcripts were developed. Smith et al. (2009) describe different techniques for developing themes. For this study, abstraction was employed as a technique that entails grouping “like with like” towards the development of superordinate themes. The criterion used in determining the superordinate themes were their relevance to answering the overarching research question.

In formulating the final superordinate theme names, the aim was to employ “both descriptive and evocative words, … [that] can guide the reader to what is essential (a deliberately phenomenological idea) about the theme” (Nigbur & Chatfield, 2025, p. 8). In this regard, Nigbur and Chatfield’s (2025) six recommendations for IPA theme naming were followed: (1) ensure theme names directly answer the experiential research question; (2) ground the names in authentic experiences rather than theoretical concepts; (3) craft concise phrases that capture the essence of the findings; (4) use metaphors or analogies to enhance relatability; (5) incorporate gerunds, particularly those reflecting how participants describe experiences; and (6) ensure the set of theme names collectively tells the story of the findings. An outline of the thematic development is detailed in Table 2.

**Table 2:** Thematic Development of Rural-Living Fisters’ Experience of Community

Initial codes	No. of participants (*N* = 40)	Emergent themes	Superordinat e themes
No local fisting peers	11	Lack of peer support	Going it alone: Self-discovery without a community
Discovering kink alone	12	Self-discovery in isolation	
No mentorship	12	Lack of peer support	
Anxiety about safety	10	Lack of guidance	
Fear of injury	11	Lack of reassurance	
No subcultural/social skills for partnered play	9	Absence of subcultural/communal education and social skills	
Long travel distances	25	Physical inaccessibility	Barriers to belonging: Difficulties in accessing community
High travel costs	22	Financial barriers	
Limited public transport	9	Limited mobility	
No local community events	16	Lack of kink spaces	
Frequent travel cancellations	10	Unreliable play opportunities	
Weather-related travel issues	5	Travel complications	
Fear of racial profiling	3	Safety risks during travel	
Feeling like an outsider	18	Exclusion from the scene	Feeling (dis)connection: Renegotiating community and kink identity
Disconnection from the city scene	14	Exclusion from the urban scene	
Resentment and frustration towards urban fisters	12	Resentment and frustration	
Limited interaction with fist-friends in community	16	Social disconnect	
Uncertainty about kink identity due to infrequent play	10	Struggle to maintain identity	
No kink-friendly doctors, nurses, or psychologists	12	Lack of medical support	Community as care: Missing support in healthcare
Fear of discrimination and avoidance of medical care	9	Fear of disclosure	
Lack of communal networks to assist with healthcare	14	Impact of the absence of community on medical and psychological health	
Online platforms as lifeline	19	Digital community	Moving online: The opportunities and challenges of online communities and connections
Virtual friendships	15	Online kink experiences	
Remote connection	14	Digital community	
Online play instead of in-person	10	Substituting digital for physical connection	
Safety challenges with apps	16	Navigating unsafety on online/apps	
No local kink / fisting spaces	20	Absence of kink venues	Longing for place, longing for playmates: The absence of communal spaces and erotic fulfilment
Fisting venues and community	12	Space and community	
Rural politics, cultural values, and commercial sustainability of kink clubs	11	Rural towns, values, space, and community	
Privacy, stigma, and the risk of exposure in rural settings	18	Privacy and stigma concerns	
Disappointing local play experiences	13	Lack of experienced partners and unsatisfying experiences	

### Researcher Reflexivity and Subjectivity

Phenomenological analysis can involve the researcher’s attempt to engage in “bracketing” (Thomas & Sohn, 2023) –a process in which they suspend or set aside their “presuppositions, biases, assumptions, theories, or previous experiences to see and describe the phenomenon” (Gearing, 2004, p. 1430). However, Smith et al. (2009) position IPA within a double hermeneutic framework, wherein the researcher is not a passive observer but an active interpreter. As Smith et al. (2009, p. 3) explain, the analytical process in IPA involves “trying to make sense of the participant trying to make sense of their experiences.” This underscores the inherently intersubjective nature of IPA, recognising that both researcher and participant bring their own backgrounds, life experiences, and attitudes to the interpretative process. At first, the participant interprets their lived experience through the account they offer in the interview and, thereafter, the researcher further interprets this account through a point of view which has itself been shaped by a review of the literature on kink and community, the strategic end goal of crafting a publishable research output, and their own personal life experiences.

This necessitated a critical engagement with how the researcher’s own desire and search for community shaped the interpretative lens they brought to the present study. Living in a country where alternative sexualities, broadly, and kink identities and practices, more specifically, remain stigmatised, even within broader sexual and gender-diverse (SGD) communities, opportunities for social and intimate connection can be significantly limited. There exist only a handful of kink and fist-friendly spaces in South Africa, and at present, no formally organized or membership-based fist-dedicated clubs or communities. Instead, the fisting community is largely constituted by informal networks of fisters and fist-curious individuals, which are predominantly concentrated in the urban and suburban areas of major metropolitan centers.

While these local networks have provided the researcher with moments of connection and affiliation, their experiences abroad have illuminated the contrast between these informal communities and the more established, visible, and organized community spaces in certain international cities. In these contexts, the researcher has experienced these communities as providing deeply affective/affecting spaces of erotic affirmation and social belonging as well as crucial resources that facilitate safer practices, social support, as well as sexual and emotional (health)care, in effect underscoring for the researcher the “limitations” of their own local context and experiences.

Thus, rather than regarding these experiences as a bias that needed to be erased, the researcher engaged in a process of research diary writing to aid in acknowledging and interrogating how their own personal experiences and preconceptions may shape the ways in which participants’ narratives were understood, analysed, and represented. One such example was the researcher’s initial tendency to hold an overly positive view of organized communities, perhaps by virtue of their own longing for such spaces. When a participant (P2) shared more critical views on these communities–describing them as “cliquish”, commercially driven, or at times exclusionary–the researcher initially found these accounts difficult to reconcile with their own idealised notions. However, through deliberate reflexivity, they sought to remain open to how participants’ experiences may diverge, recognising that while organized communities can offer vital support, they are not without their own internal hierarchies and exclusions.

## Results

The IPA rendered a total of six main themes: (1) the experience of isolation in the formative journey of self-discovery; (2) geographic, logistical, and financial difficulties in accessing community; (3) feelings of (dis)connection from the community and the struggle for kink identity; (4) the absence of community networks and resources in supporting healthcare needs; (5) the opportunities and challenges of online communities and connections; and (6) the lack of communal spaces and local networks of partners in fostering sexual and erotic satisfaction. Each of these themes is outlined in the sections which follow.

### Theme 1: Going It Alone: Self-Discovery Without a Community

For participants who discovered their interest in fist-play while living in rural and remote areas, their formative journey into fist-fucking was often marked by the absence of community. Unlike those who came into their kink while residing in urban or metropolitan centers, these participants experienced a distinct lack of in-person communal support during their early solo-play and exploration. For these participants, community was constructed as a vital psychological and social support structure, and its absence was recurrently described in terms of both a sense of personal isolation and longing for the mentorship and peer guidance believed to be provided by fisting communities:

It wasn’t ‘til I went to Tokyo to work for a few years that I finally met others into fisting. Back home, it was impossible. No community, no playmates, just me and my fantasies. (P14, 28, EA, Versatile, Rural, Japan)

I’m always struck by guys who grew up in cities and found their way [into fisting] with the help of a whole community. Friends, mentors, [and] partners, there’s so many people who played a role in supporting their journey. I didn’t have that and there’s always been a part of me that feels like I missed out. I think for a lot of us who grow up in the middle of nowhere it’s a lonely road. (P18, 33, B/A/AD, Versatile, Rural, Canada)

In this theme, the absence of community was often characterised by experiences of fear, uncertainty, and a sense of ‘missing out’ on communal support and knowledge for participants. In addition to feelings of social and sexual isolation, some participants also described how this lack of community led to uncertainty about their own capability to engage in fisting safely and appropriately. While some turned to online platforms such as websites, discussion forums, and fisting pornography to educate themselves in their early self-discovery, the absence of a network of peers and mentors underscored for these participants not only the idealised role of community as an essential educational and socialising resource but, also, an idealised trajectory of kinked and subcultural identity development, as a part of which kink communities were perceived to play a central role. For these participants, community was not only a site of erotic connection but also a means of developing the personal confidence, subcultural knowledge, and social skills necessary to play with other fisters:

There was no one to talk to about it, so self-play became my way of figuring things out. […]. I wanted to explore more but there was also, I guess, doubt and anxiety. I was afraid that if I hurt myself, I would be incontinent for life. No one was there to reassure me. […]. Not having access to a tribe of people like me who could normalize my desire and my doubt was a real challenge. (P20, 46, M/M, Bottom, Remote Rural, USA)

I had really no confidence in the first year I started to put myself out there. I had never played with anyone before, I felt I would be hopeless in a social setting. If I look back now my feeling is that it would have been easier for me if I was able to start off in a community. (P38, 28, W/E/C, Top, Remote Rural, France)

I knew I could take my own fist, but could I take someone else? I knew how to douche and prep when I played by myself, but was that good enough for someone else? You can learn the basics on your own, but how to speak and act and play with another fister is what a community can teach you. (P25, 40, W/E/C, Bottom, Remote Rural, USA)

### Theme 2: Barriers to Belonging: Difficulties in Accessing Community

For most participants, the experience of community was often framed in terms of the difficulties in accessing playmates, clubs, and networks of fisters in person. Physical distance was frequently cited as a key barrier to participation in community events. Many described how living in rural areas meant that the nearest fisting communities were concentrated in urban centers, requiring significant travel. However, participants also drew attention to ways in which geographic distance was often coupled to and exacerbated by other financial and logistical burdens of travelling long distances, including the cost of transport, time constraints, the costs of accommodation if they needed to overnight, and the expense of travelling on their overall budget and cost of living. These combined factors reinforced a sense of exclusion from community spaces and networks:

I feel locked out the community. I’m 360 miles [579 km] from any [community] space or network. That’s a 6-hour drive. That physical distance is a barrier and there’s basically no way for me to get over that. My car ain’t gonna get me that far and, even if it could, I couldn’t afford the gas for that. (P29, 29, W/E/C, Top, Rural, USA)

Because of how far I have to travel I’ll spend more time on the road trying to reach a scene than I do playing. I have to evaluate if that’s worth it. That’s a terrible fucking position to be in. I need to evaluate if the expense of traveling outweighs the joy of connecting with someone. (P35, 31, W/E/C, Bottom, Rural, Australia)

I literally have to do a cost-benefit analysis. Like, is spending money to travel going to impact my ability to pay rent. … [I]f I spend the money on a bus ticket, then I’m gonna need my playmate to host me because I can’t pay for the bus and accommodation. Guys don’t always sign up for that. They’ll let you cart your ass all the way there but they don’t really want you sticking around for a couple days after you’ve played. (P9, 34, W/E/C, Bottom, Remote Rural, USA)

For those participants who managed to travel long distances to participate in play scenes, additional challenges often arose. Participants spoke about the unpredictability of meetups, particularly last-minute cancellations or unexpected changes, which exacerbated the emotional and financial investment involved in seeking out play partners and community:

People can be flakes. I’ve been in situations where I do all the prep work, I drive 10 hours to Toronto and literally 15 minutes before the meet-up I get cancelled on. No excuse. No apology. It’s soul-destroying. (P34, 32, B/A/AD, Bottom, Rural, Canada)

There’s always some anxiety about how I’m going to feel by the time I get there. I’ve left my place feeling great and then had a haemorrhoid pop up out of nowhere on the road and had to cancel at [the] last minute. (P6, 27, ME/NA, Versatile, Semi-Rural, Germany)

I’ve travelled 200 kms to meet a partner and after getting there I just can’t get a good cleanout so play is off the table. For a bottom who lives close by, maybe that’s not too bad because they can always reschedule for the next weekend. I don’t have that luxury. (P11, 30, W/E/C, Bottom, Rural, Sweden)

Beyond logistical and financial hurdles, broader social and environmental factors also shaped rural-living fisters’ ability to access playmates and community events. Some participants recounted experiences of racial profiling, law enforcement harassment, and unexpected weather-related obstacles, all of which added layers of difficulty in travelling to urban centers for play:

… [T]his far north, summer is only a few weeks. I have to wait for that 3-week window every year because winter brings challenges. In summer I can get to Oslo in 20 hours. But for most of the year there can be such bad snowfall or ice on the [railway] tracks that it can cause really long delays and mess up any plans I make in Oslo. (P12, 39, W/E/C, Bottom, Rural, Norway)

… I’m black in the South. That means I can get pulled over for anything. I’ve been pulled over tons of time driving down to New Orleans. The cops here can pull you over anytime and search you if they want, especially if they pick up that you’re gay and they want to give you a hard time. It’s happened before. A cop searched my bags, found my toys and started to get very abusive. (P26, 43, B/A/AD, Bottom, Rural, USA)

Within this theme it is worth noting how P26’s account underscores their racialised subjectivity and positionality as “Black in the South” and how this introduces an additional barrier to travelling and accessing urban fisting communities. This account highlights that community access is shaped not only by geography and economic factors but also by racialised, homophobic, and kinkphobic norms and policing practices that disproportionately target kinksters of colour.

### Theme 3: Feeling (Dis) Connection: Renegotiating Community and Kink Identity

Many participants described experiencing both connection and disconnection from the broader kink community. While they identified as part of “the” fisting community, their geographic isolation and infrequent play opportunities with ‘a’ specific local fisting network or group of playmates often informed an ambivalent sense of belonging. Across the accounts of this theme, participants’ experiences of (dis)connection took different forms. Some participants expressed a feeling of belonging in principle but not in practice, as their physical distance from play spaces and other fisters rendered their sense of connection abstract rather than tangible.

For many participants, their (dis)connection was a distinctly bittersweet experience: while they found joy in seeing the fisting community grow both globally and, in some cases, locally, their physical remoteness left them feeling excluded from this growth:

Um, so, I know I’m part of *the* community, I’m just not *in* it. If that makes sense? … I’ve wrestled with this feeling for the 10 years I’ve been on [Isle of] Skye. I feel the connection in my heart, just not always in my hole. (P36, 50, SA, Bottom, Rural, UK)

There’s a weird disconnect … like I’m observing it all from a distance, but can’t quite be a part of it. I’m happy to see how the community is growing, even in Japan, but I’m excluded from that because I live so far away from it. Yes, it’s bittersweet. (P15, 42, EA, Bottom, Rural, Japan)

For other participants, their (dis)connection to the fisting community became increasingly shaped by feelings of jealousy and resentment, particularly when attending kink events or city-based play spaces. Seeing other fisters plan and engage in regular play underscored for them the stark disparities in accessing community between urban and rural kinksters. The inability to build lasting play connections added to their frustration, as potential partners often moved on due to the logistical difficulties of maintaining long-distance play relationships:

If I go to Syd[ney] for a party I’m obviously excited ‘bout it but there’s always some jealousy underneath. You’ll hear people talk about who they’re going to play with next week, and the week after that, and I’ll feel jealous because that’s not an option for me. It’s so easy for them to make plans and then make it happen. (P19, 37, SEA, Bottom, Remote Rural, Australia)

I’ve met some great playmates who I’d like to play with again, but telling someone you can only play again in another 6 months doesn’t fly. Guys move on ‘cause they’ve got a lot more options where they are. I think it’s natural to feel resentment because of the situation I’m in. Resentment is what I feel most and it doesn’t feel great. (P3, 29, W/E/C, Bottom, Rural, Australia)

Even when participants were able to attend events, their infrequent participation sometimes left them feeling like outsiders, struggling to integrate into the established social circles of urban fisting networks:

If I do go [to an event] then it sometimes feels like I have to start from the beginning. I get treated like the unknown quantity because I’m not around as much. Communities can be great but they can also be cliquey and inaccessible. (P13, 52, W/E/C, Bottom, Denmark)

I was at a party in Berlin last year and I told a guy where I was from and then he started calling me ‘Mr. Countryside.’ It was meant as a joke, but his attitude made me feel like I didn’t belong there. I just said, ‘I don’t need to live here to be part of this.’ Just showing up, playing on my terms. That was me claiming my place. This community is mine too. (P30, 38, W/E/C, Versatile, Germany)

For some participants, the disconnection from their community was most deeply experienced in the social dimension of their kink. Beyond just a space for intimate play, many participants described their kink communities as networks in which meaningful social bonds, friendships, and a sense of belonging were developed. Without these networks, participants described a profound sense of isolation, missing not only sexual connection but also the camaraderie, shared understanding, and support that come with being part of a kink-identified community:

I think for most kinsters, community is a big part of the lifestyle. There’s social connections and friendships, on top of the sex. Depending where you’re at in your life those [social] connections actually come to mean a lot more. Since I moved to Lüderitz, I’ve really missed that. (P37, 44, W/E/C, Bottom, Rural, Namibia)

For some participants, their sense of disconnection also contributed to an evolving uncertainty about their kink identity. Limited opportunities for participation in partnered play and other community events raised difficult questions about the legitimacy of their identity as a fister, particularly during extended periods without contact with other fisters:

There’s that expression about a tree falling in a forest. If no one hears it, does it make a sound, you know? I go through something similar. If I haven’t played for a while, am I still a fister? Am I still a fister if I’ve just been sitting on toys for a month? (P22, 47, ME/NA, Bottom, Rural, Germany)

Self-play, toys get you by, but it’s not the same as being with someone. […]. There’s a rhythm when you’re with someone who’s reading your body, adjusting, pushing, holding back. Toys don’t do that. It’s missing … the connection. Being with a partner, feeling that shared intensity, … is what makes fisting feel like something I am, not just something I just do. Without that, it’s easy to feel like I’m just going through the motions, rather than really living it. (P39, 30, L/H, Versatile, Rural, Brazil)

### Theme 4: Community as Care: Missing Support in Healthcare

Several participants reported significant challenges in accessing kink-affirming healthcare in their rural locations. Many of them described a reluctance to seek medical assistance due to fears of judgement, stigma, and a lack of specialist knowledge among local healthcare providers. This hesitancy was however compounded by the absence of local kink communities that might otherwise serve as informal networks for sharing advice, recommending trusted healthcare professionals, or offering practical support in times of need. In comparing their situations to those of their urban counterparts, who were perceived to have access to referral networks within kink communities, participants described feeling isolated when navigating healthcare concerns. Without a trusted local network, they were left to manage injuries or complications alone, often relying on online resources or self-care rather than seeking professional medical intervention. This lack of communal support heightened some participants’ anxiety about seeking medical care, particularly when dealing with prospective injuries related to fisting:

Fisters talk. In the cities, you share information about good doctors who are open minded and will treat you with respect. In the sticks, you don’t get that. There’s no referral network you can access to ask for help or which doctor to see. (P33, 36, W/E/C, Bottom, Remote Rural, USA)

If I picked up an injury and only realised [it] when I got home, I’m not sure who I could go to. The doctors are pretty conservative here and there’s no community to fall back on either. I once tried talking to one [doctor] about a [anal] fissure I got from some rough [receptive anal] sex and all I got given was a lecture, not medical care. (P21, 35, W/E/C, Versatile, Rural, USA)

For some participants, the problem was not only stigma but also the sheer lack of healthcare infrastructure. In cases of serious injury, the absence of nearby kink community members who might otherwise provide support intensified feelings of vulnerability:

P31 (27, L/H, Bottom, Rural, Brazil): There’s one clinic in my town. There’s no hospital and no ambulance service. I would rather be safe than sorry. If I was hurt seriously, there’s no other fisters I can call for help. I would probably have to drive myself to a hospital.

Researcher: How far is that?

P31: Maybe 2 hours. In Manaus.

The experiences shared by participants across this theme highlighted how geographic isolation not only limited their access to kink communities but also restricted crucial healthcare resources. Without accessible communal networks to provide guidance, referrals, or emergency support, participants often experienced a compounded sense of risk that included both physical safety and the psychological toll of navigating healthcare in a potentially hostile environment.

### Theme 5: Moving Online: The Opportunities and Challenges of Online Communities and Connections

In this theme, online spaces were often described by some participants as a crucial lifeline, offering a means to connect with others who shared their kink. Without physical access to fisting communities, virtual interactions become a way to maintain a sense of belonging and engagement. Online platforms such as chat rooms allowed for the exchange of knowledge, support, and even play strategies, creating new opportunities to reconfigure community in and through digital space:

The internet is like a lifeline in the desert. Without a local community, it’s my only way to connect and stay part of the scene. (P24, 49, W/E/C, Versatile, Semi-Rural, New Zealand)

Actually, I kinda feel more connected to [other fisters] than I ever did when I lived in San Francisco. My [Autism Spectrum Disorder] always made it awkward for me to interact with other people. But, online, I’m a lot more comfortable and despite being so isolated, I’ve probably got more [online] fist-friends now than ever. (P25, 40, W/E/C, Bottom, Remote Rural, USA)

For some participants, embracing virtual spaces as a substitute for in-person play proved an essential component of adapting their sex/uality to rural life. Though physical encounters were less frequent, participants described the development of creative strategies that enabled them to maintain intimacy and connection with other members of the fisting community, including video calls, chat rooms, and media exchanges:

I actually thought I was going to struggle when I left Chicago but I’ve managed to set up a really nice community of playmates online. Even though I can’t play [in person] as much as I would like, I’ve adapted. Chat rooms, video calls, and swapping clips with other [fist-]pigs. It’s my new normal. (P17, 44, W/E/C, Bottom, Remote Rural, USA)

Although online communities provided opportunities for virtual connection, many participants acknowledged that they did not fully replace the experience of in-person partnered play. The absence of direct corporeal connection, concerns over trust and safety, and the difficulty of vetting potential partners presented unique challenges for rural-living fisters. Unlike their urban counterparts, who often relied on shared networks to verify a prospective playmate’s reputation, participants attested to feeling left to the challenges in assessing a partner’s experience, expertise, or intentions solely through dating apps or websites:

The benefit with being in a community is that you have a network of buddies you trust and have no problem playing with. The downside here is I don’t have that. I have to rely on what drifts through on Grindr and you can’t always trust what you find there. Even if you find them on Recon. Someone might claim to be this experienced top, but there’s no real way to verify that. (P2, 45, B/A/AD, Bottom, Rural, USA)

Safety is always at the front of my mind because it’s not like I can just ask someone who they’ve played with and check with that person to [cross]check the story. (P32, 48, W/E/C, Top, Remote Rural, Sweden)

### Theme 6: Longing for Place, Longing for Playmates: The Absence of Communal Spaces and Erotic Fulfilment

Participants highlighted the absence of physical kink spaces as a significant barrier to both scenes of play as well as consolidating more local and accessible community networks. Within this theme, some participants described an interconnection between communal fisting spaces and the sustainable formation of fisting communities. In one such account, P28 highlighted how the interconnection between space and community was visible both historically and contemporarily:

Our community was really anchored by clubs like Mineshaft, in New Work. Even today, in London or Berlin there are fetish bars and clubs that anchor those communities. These spaces, especially for fisters, are the lifeblood of our community. The fisting communities there thrive and grow because there’s a space where fisters or fist-curious people can come together, play and socialise. The absence of a community means that spaces dedicated to fisting are just not viable. But, it’s also the absence of those spaces which means a community probably doesn’t have a chance to get a foothold. (P28, 51, M/M, Bottom, Semi-Rural, UK)

Economic and cultural factors were however frequently cited by participants as obstacles to establishing kink-friendly venues in rural areas. Moreover, the lack of dedicated kink spaces meant that play often had to take place in private homes, which in itself raised logistical and social challenges related to privacy, discretion, and the risk of discrimination for rural-living fist-fuckers:

The lack of a space is understandable. There’s obviously a commercial consideration. There’s no client base to make that a sustainable business. There’s also a different politics outside of big cities [in the USA]. Can you imagine someone trying to open a fetish club in Jetmore! No one on the town council would give permission. We have more Churches than hospitals. That tells you a lot about the people and the politics. (P5, 50, W/E/C, Top, Remote Rural, USA)

There’s no local [kink] spaces so you’re having to play at people’s houses. This brings its own complications. In small towns, everyone knows everyone else’s business. There’s not a single house which has a fence here. If there was a bunch of cars parked outside my place for a night with thumping music and some audible moaning, I think people would start talking. Hell, they’d probably call the cops on me. (P4, 38, B/A/AD, Bottom, Rural, USA)

Even when gay bars or venues existed in their town or nearby towns, participants noted that these spaces were not inclusive of kinksters, further reinforcing feelings of exclusion and limiting opportunities for community building:

We’ve got a small gay bar [in our town], but the vibe is very Log Cabin [Republican]. It doesn’t really accommodate queer expressions of what it means to be gay. It’s pretty masc for masc and in no way is it kink friendly. (P1, 32, W/E/C, Bottom, Semi-Rural, USA)

For some participants, the absence of local spaces in which fisters could meet and play meant they had to rely on the willingness or curiosity of partners they met locally through dating apps to experiment with fisting. However, the lack of fisting experience and training among such potential partners typically resulted in unsatisfying encounters. Many participants expressed persistent frustration over the inexperience of local partners, which diminished the quality and safety of their play and forced them to adjust their expectations regarding erotic and sexual fulfilment:

I’ve met a few local guys who are open to experimenting with fisting but none of them has ever been a satisfying experience. Like, if they want to bottom, they’re not always properly trained to take a fist or, if they want to top, they’re so new that I don’t trust them enough to play with me. (P7, 41, L/H, Bottom, Rural, USA)

In hindsight, I didn’t realise that moving to Springbok would mean mourning the community I lost *and* this important part of me. I’ve had to be resilient enough to know I’ll never be completely satisfied. My desires are never going to be met the way I need them to be as long as I live here. (P16, 31, W/E/C, Bottom, Remote Rural, South Africa)

## Discussion

The findings of the present study extend existing literature on communities of kink by foregrounding the lived experiences of community for rural-living fist-fuckers. In a body of scholarship that tends to privilege urban, connected, and visible kink communities, rural-living kinksters remain located at the periphery of analytical attention. Although existing research has proven important in advancing an understanding of kink communities as socially supportive and sexually affirming systems within which kinksters fashion meaningful identities, this study highlights how the erotic, social, and geographic dislocation from the communal networks and spaces of metropolitan areas can underpin contrasting experiences in how community is understood, longed for, and, at times, creatively reconstituted for rural-living kinksters. Participants’ accounts reveal that rurality may both constrain and reconfigure possibilities for community, belonging, and satisfaction. These findings demonstrate that rural-living fisters undertake a complex (re)negotiation of community connectedness, characterised by a multiplicity of sometimes competing experiences, including isolation, desire, loss, adaptation, and resilience. If community is so central to the exploration and formation of a kink identity, as existing research suggests, then the findings of this study offer an opportunity to critically rethink dominant, metronormative assumptions about where and how kink communities are formed, maintained, and experienced, foregrounding instead the uneven geographies of access, the improvisational labour of connection, and the diverse ways that kink community is imagined and enacted beyond the urban center.

Echoing existing research (Vivid et al., 2020), the findings of this study affirm the crucial role that community plays in supporting kink identity development (P20), sexual education and health (P21), and socialisation and acculturation to subcultural norms and practices (P25). As Weiss (2011, p. 12) argues, “kinky people become real…practitioners through participation in a social, sexual, and educational community,” and the experiences shared by rural fist-fuckers reiterated this protective and developmental function. Yet the findings also underscored how the absence of community, particularly during the formative stages of sexual self-discovery, resulted in feelings of isolation, uncertainty, and challenges in kink identity formation. Sprott et al., (2019, p. 50) conceptualize the kink community as a “community of practice that is built on joint activities and sharing of information, wherein people build relationships…to help each other in learning or skill acquisition.” For rural fisters, limited access to such networks and spaces meant navigating their kink largely in isolation, often without guidance on safety and technique. These findings highlight that for fisters, community is not merely a desirable support system, it forms an alternative infrastructure of care and support through which their identities are made personally intelligible and intimately viable.

While this study set out to develop a more capacious account of kink community by centring the experiences of rural-living fist-fuckers and challenging the dominance of metronormative narratives, it is notable that participants did not describe the formation of in-person, place-based rural kink communities. Instead, they spoke overwhelmingly of their disconnection from urban kink spaces and the absence of locally grounded networks of playmates. This stands in contrast to studies of non-kinky SGD individuals, which have shown how rural-living SGD people often resist “compulsory metronormativity” (Herring, 2010, p. 75) by forging meaningful and even thriving rural lives and communities. By contrast, what emerges in this study is that urban fisting communities continue to function as the sexual and social gravitational center for rural-living fist-fuckers. While this may not directly subvert metronormativity, participants’ accounts nonetheless expose its workings: how it structures access, shapes expectations, and conditions affective experiences of belonging and exclusion. The reasons for this may lie in the socio-political and economic contexts of rural life, which render kink community building and subcultural placemaking risky or unfeasible (as noted by P1, P4, P5). Yet they may also be particular to the practice of fisting itself. As Martin (2025b, p. 1124) observes, “unlike other forms of kink that create sensory stimulations that are registered on or just beneath the surface of the skin…[t]he erotic work of fist-play principally takes place inside the receptive playmate’s body.” As such, the meanings that fisters ascribe to their identities, values, and practices are deeply rooted in the experiences of trust, intimacy, and what P39 described as the “shared intensity” of fisting borne through direct scenes of interpersonal contact and play. Perhaps most evident in P22’s rhetorical question: “Am I still a fister if I’ve just been sitting on toys for a month?”; this uniquely relational and inter-corporeal mode of erotic meaning-making may render fist-fuckers especially dependent on in-person communities that, for the most part, remain spatially concentrated in urban centers.

The premium placed on physical connection with other fisters as a defining feature of community was not only articulated through rural participants’ longing for knowledgeable, skilled, and trustworthy playmates to be in closer proximity (P7, P16), but also expressed as an experience felt and lived through their bodies. As P36 remarked, “I feel the connection in my heart, just not always in my hole.” This underscores how community connectedness becomes embodied and enfleshed through the sensations and sensualities of fist-play. For rural-living fisters, community is not only about shared identity; it is also about the pleasures which sensually materialise and corporealise this connection and which are, for many of them, achingly absent in remote settings.

In exploring rural-living fisters’ experiences of community, the findings of this study also drew attention to the diverse and contradictory ways that community was understood, felt, and navigated. As Jones (2020, p. 123) remarked in their study of the rope bondage practitioners, “[c]ommunity is a contested concept.” In the same way, community was not a static idea expressed by participants, but a shifting discursive formation. On one hand, community articulated a spatially bound material reality defined by spaces, networks, and other bodies (P28). On the other, it was deployed to invoke an imagined community or “tribe” (P20) that described an abstract sense of fictive kinship. However, across these discursive designations, participants recurrently conveyed the deep affective stakes of community: the comfort, recognition, and safety it offers in its presence, and, as P3 and others described, feelings of longing, alienation, and loss in its absence.

Pennington (2018, p. 62) contends that kink “communities create a sense of affective belonging.” This affective connection to community operates not only in facilitating the felt bonds of attachment that support community affiliation, but also in underpinning feelings of sexual citizenship wherein belonging is entangled with recognition, legitimacy, and the right to participate. This was particularly evident in the way P30 not only felt alienated by being referred to as “Mr. Countryside” but, in response, their need to reclaim their place within the urban scene and assert their belonging, visibility, and sexual citizenship. Their communal citizenship, then, was not defined by proximity to urban kink spaces, but by their affective and discursive claim to the community, in effect challenging the spatial hierarchies that often privilege urbanity as the locus of legitimacy and belonging. Moreover, this suggests that affect remains a powerful lens through which to theorise kink sexual citizenship and community, as it moves beyond narrowly identitarian claims of similarity or difference to consider the emotional, relational, and spatial dimensions through which communal belonging is lived and felt (Pohtinen, 2017).

The experiences of community presented in this study also demonstrate how rural marginality can be shaped and reinforced by racialised geographies. Unlike many other participants, whose difficulties in accessing community were often financial or logistical, P2, P4, and P26, all of whom identified as B/A/AD and resided in the US, described racialised experiences of discrimination that introduced an additional layer of precarity when travelling to access urban kink spaces or events. Notably, P26 recounted being routinely pulled over and subjected to invasive searches while travelling, a pattern that illustrates the compounding effects of multiple-minority stress (Cyrus, 2017). For these rural-living fisters of colour, the intersection of racialised subjectivity, sexual orientation, and kink affiliation intensified the risks associated with mobility and public visibility in the rural US. While several important studies have shown that the experiences of exclusion among kinksters of colour are informed by their ethnic or racialized identities (Cruz, 2016; Erickson et al., 2022; Liang, 2022; Williams & Sprott, 2022), little attention has been paid to the specific burdens entailed in travelling to urban kink communities from rural areas these kinksters. What this study brings into sharper focus is that, for some rural-living fisters of colour, travel is not merely a logistical challenge, but a mode of movement fraught with compounded risks, surveillance, and the threat of harassment. This is especially in contexts where mobility has been increasingly securitised, creating new forms of (racially selective) mobility policing (Adey, 2009; Pallitto & Heyman, 2008). These experiences underscore the spatial and racial politics of kink participation: while rural-living white kinksters (or kinksters in more ethnically or racially homogenous countries) may access community through travel with relative ease, kinksters of colour must navigate landscapes where the very act of movement can trigger punitive responses (Jefferson-Jones, 2021; Selod & Rosenthal, 2025). As such, the ‘freedom’ to access kink community is not equally distributed. It is shaped by rural racisms and racialised systems of mobility control that tend to render Black, Brown, and queer bodies, hyper-visible and vulnerable in ways others are not (Browne, 2015; Cloke, 2006).

While rural isolation from in-person kink communities posed significant challenges for many participants, others found alternative ways to reimagine and reproduce fisting community through digital and virtual means. Reflecting research on the coping strategies employed by non-kinky SGD individuals in rural areas (Cody & Welch, 1997; Preston & D’Augelli), several fist-fuckers (P16, P22, P39) described practices of mental and erotic resilience that helped sustain both their sexual identities as fisters and their erotic satisfaction, such as self-play and the use of toys. Others (P17, P24), turned to the internet to reconstitute a sense of sexual and social community, engaging online platforms as spaces for dialogue, fantasy, and digital intimacy and exchange that creatively reshaped their communal connectedness. These practices echo existing literature on how digital spaces function as a virtual infrastructure for kink communities (Wignall, 2022), particularly by decoupling access to community from the geographic proximity and urban concentration often presumed necessary. These virtual possibilities even afford a new opportunities for more accessible communal connection. P25, who identified as having Autism Spectrum Disorder, described in-person interactions as “awkward,” while finding online interactions more comfortable. While P25’s experience may be unique, it nonetheless highlights how the urban epicenter of kink belonging can be displaced through new forms of technological and “digital sexual placemaking” (Sundén et al., 2022, p. 436), offering alternative routes for rural-living fisters to cultivate community. This shift also signals a subtle resistance to the kinknormative pressure to be embedded within urban communities, a pressure implicitly and explicitly echoed by many participants.

Despite the creative affordances of virtual connectivity, rural realities still contour digital experiences for rural-living fisters. Geolocation-based apps like Scruff and Recon, for instance, were often described as less effective in remote areas due to limited nearby users, while several participants (P2, P32) raised concerns about personal safety when arranging online-initiated meetups. This suggests that while the internet may collapse geographic distance, the digital technologies can present a paradox for rural-living fisters: even as they expand communal horizons, they remain tethered to the material and cultural conditions of rural life and space.

Finally, it is also worth noting that digital community does not necessarily diminish a yearning for physical spaces among rural-living fisters. Dedicated communal spaces offer fisting communities the opportunity “to get a foothold” (P28), underscoring how place can be critical to enabling social connection and community building. Such spaces matter not only as physical places for gathering and play but also as erotic archives that act as repositories for the shared history, intimacy, and identity of fisters. Venues like The Catacombs and Mineshaft, along with more contemporary spaces, come to hold a special import in the subcultural imaginary of fisters. They are symbolic and material places of communal belonging and memory. The longing for these kinds of spaces is, at least among some of the rural-living fisters in this study, a desire for a materially grounded sense of community and connectedness that digital forums, however rich, may not always fully replicate.

### Limitations

There were some limitations to the present study. A key challenge in the initial conceptualization of the study was in establishing how the rurality of participants’ and their living situations would be determined. Rurality is often understood through objective markers, such as (but not limited to) geographical location (e.g. proximity to the nearest urban center), population density (i.e., the number of people per square kilometre), or the degree of access to infrastructure and services (Fischer et al., 2024). While these criteria may offer a more measurable indication of participants’ living circumstances, as a phenomenological study, the focus of this research was not on the objective criteria of rurality but on participants’ lived experiences of rurality and how this informs their subjective sense of physical, psycho-social, and identity connection to the/a fisting community. Given the focus on participants’ experiences of kink community, the study ultimately opted for a determination of rurality that relies heavily on participants’ perceived remoteness to fisting spaces, resources, and in-person play opportunities. While this was useful in centring participants’ own perceptions of their access to kink communities, a limitation is that it may conflate rurality with remoteness, the latter of which may be more predicated on the degree of isolation rather than strictly on geographic or infrastructural factors of rurality.

Following from this, a further limitation was that the categories offered to participants for them to define and classify their rurality, may lack nuance in capturing the full range of what rurality encompasses. Rurality, especially for SGD people, is a complex, multifaceted identity status and socio-geographic positionality (Forstie, 2022; Thomsen, 2021). It can be influenced by social, cultural, and economic factors as well as the relative prospect of violence and discrimination that may not be fully reflected in the categories participants were asked to use. An alternative approach could have been to invite participants to describe in their own words what they perceived as making them, or their living situation, rural. Analysing these participant-produced descriptions might have offered a deeper understanding of how each individual self-defined their rurality, providing a richer view of the diverse experiences of rurality within the sample.

Furthermore, the study explicitly focused on recruiting individuals who identified as fisters and reported a subjective sense of affiliation with the/a fisting community, while excluding those who engaged in fisting without broader community ties. The exclusion of rural-living individuals who fist but do not identify with any kink community may limit the study’s ability to capture the full spectrum of experiences in rural areas. These individuals may navigate their identities and sexual practices in ways that differ significantly from those who derive their kink identity from community connection. As a result, the findings presented here overlook the challenges, strategies, and meanings that non-affiliated fisters ascribe to their practices. This reflects a broader trend in the study of alternative sexualities, where research tends to over recruit and overrepresent kink-identified individuals with established community ties (McCormack et al., 2022; Wignall, 2022).

### Conclusion

This study has foreground the often-overlooked subject of community for rural kinksters by exploring the experiences of a multi-national sample of 40 fist-fuckers living in rural and remote areas. The study offers insight into how these fist-fuckers negotiate community and, with this, navigate their kinked identity, practices, and relationships in contexts marked by geographic isolation as well as physical and cultural constraints. Taken together, these circumstances limit rural-living fist-fuckers’ access to partners, networks, and spaces of in-person play, social interaction, and (health)care. The findings present a portrait of community that remains largely metrocentric, with urban fisting scenes continuing to occupy the center of subcultural discourses on belonging. In this regard, many rural-living fisters expressed feelings of disconnection, marginalisation, and longing, underscoring the emotional, social, and erotic toll of rural marginality. However, the findings also suggest that rural-living fisters can forge alternative configurations of community through digital and online modalities that challenge metronormative logics and expand how they understand and experience their own sexual citizenship and communal kinship. While communities of kink may themselves exist on the margins of normative sexual culture, they too can reproduce hierarchies that center urbanity and metropolitan belonging (Bennett, 2025). This study invites further research into the lives of rural kinksters and, in particular, how they (re)imagine and (re)make community at the margins, as well as remake the very margins of what is understood to constitute community.

## Data Availability

Not applicable.
